# Should zygote intrafallopian transfer be offered to all patients with unexplained repeated in-vitro fertilization cycle failures?

**DOI:** 10.1186/1757-2215-7-7

**Published:** 2014-01-20

**Authors:** Itai Gat, Jacob Levron, Gil Yerushalmi, Jehoshua Dor, Masha Brengauz, Raoul Orvieto

**Affiliations:** 1 Department of Obstetrics and Gynecology, Chaim Sheba Medical Center (Tel Hashomer), Ramat Gan, Israel; 2 Sackler Faculty of Medicine, Tel Aviv University, Tel Aviv, Israel; 3 Pinchas Borenstein Talpiot Medical Leadership Program, Chaim Sheba Medical Center (Tel Hashomer), Ramat Gan, Israel

**Keywords:** ZIFT, IVF, Repeated implantation failure, Pregnancy rate

## Abstract

**Background:**

One of the suggest strategy for patients with repeated implantation failure (RIF) is zygote intrafallopian transfer (ZIFT). However, no data exist regarding to the issue of when and under which circumstances should ZIFT be offered to patients with RIF? We therefore aimed to examine whether repeated implantation failure (RIF) patients characteristics or their previous controlled ovarian hyperstimulation (COH) variables may differentiate between those who will conceive following a ZIFT cycle and those who will not.

**Methods:**

Forty seven consecutive women admitted to our IVF unit during a 7 year period, who underwent ZIFT for RIF, were included. Ovarian stimulation characteristics, number of oocytes retrieved and number and quality of zygotes/embryos transferred were assessed and compared between the ZIFT cycle and the previous IVF/ICSI cycle and between those who conceived following the ZIFT cycle and those who did not.

**Results:**

Twelve clinical pregnancies (clinical pregnancy rate- 25.5%) were recorded following the ZIFT cycle. Those who benefit from ZIFT were young patients (≤31 yrs), who underwent ≤6 cycle attempts, yielding over eight 2PN embryos with low (≤0.4) ratio of number of top-quality embryos to total 2PN embryos. Moreover, in those destined for a ZIFT cycle, only those with >7 2PN embryo should undergo a transfer of at least five 2PN embryos.

**Conclusions:**

Further large prospective studies are needed to identify the specific characteristics of RIF women who may benefit from ZIFT.

## Background

Controlled ovarian hyperstimulation (COH) is considered a key factor in the success of in vitro fertilization-embryo transfer (IVF-ET) [1]. However, despite the great improvements in ovarian stimulation protocols and fertilization procedures, implantation rates per embryo remain at approximately 15% and many couples are still left frustrated following multiple failed attempts [2].

While analyzing a data from 78,325 cycles in 34,430 women registered in the Human Fertilisation and Embryology Authority data base, Templeton and Morris [3] found that four or more previous IVF cycle attempts significantly reduced both the odds of a birth and the odds of multiple births. Meldrum et al. [4] analyzed the data from the 1994 ASRM/SART registry and found that cycle rank 4 or more demonstrated a significant reduction in delivery rate. Moreover, while combining their results with those of previous studies, they demonstrated a modest reduction of successful outcome with increasing previous cycle failures. Recently, in an attempt to examine whether and when conception may be achieved in multiple repeated IVF cycles, we conducted a survey of the outcome of IVF in 2760 consecutive cycles in our unit. In accordance with the aforementioned studies, we also observed a significant decreased in pregnancy rate beyond the 3^rd^ IVF cycle attempt, with no significant decrease between the 4^th^ to the 20^th^ attempt [5].

In couples with repeated implantation failures (RIF)- those after failure of three cycles in which reasonably good embryos were transferred [6], a comprehensive investigation and many therapeutic strategies were offered. Counseling couples with RIF remains, therefore, one of the most frustrating, but rather challenging issue for the fertility specialists. While many strategies were offered for the treatment of RIP, including hysteroscopy, endometrial injury, change in the stimulation protocol, blastocyst transfer, assisted hatching, preimplantation genetic screening for aneuploidy, etc. [6-8], no compelling advantage for one protocol over another has been hitherto established [6].

One of the suggest strategy for patients with RIP is zygote intrafallopian transfer (ZIFT) [9-13]. This procedure, that was primarily advocated for non tubal factor infertility, offers the appropriate physiological environment for the growing zygote/embryo (avoiding the in vitro culture systems), with an optimal synchronization between embryonic and endometrial development. Moreover, since it prevents intrauterine manipulation and the consequent possible embryo expulsion secondary to sub-endometrial/myometrial contractions, it was also offered to patients with RIF.

Prompted by these findings, we sought to examine whether patients characteristics or previous COH variables may differentiate between those who will conceive following a ZIFT cycle and those who will not. This will aid both fertility specialists’ counseling and their patients in adjusting the appropriate treatment strategy for RIP.

## Methods

We reviewed the computerized files of all consecutive women admitted to our IVF unit during a 7 year period, who reached the ovum pick-up (OPU) stage. Only patients undergoing ZIFT were included. The study was approved by our institutional review board.

All the usual indications for IVF/ICSI and accepted protocols for ovarian stimulations were included. The selection of type of COH protocol used was the decision of the treating physician and largely dependent on the fashion at the time. In all protocols, gonadotropins were administered in variable doses, depending on patient age and/or ovarian responsiveness in previous cycles, and further adjusted according to serum E2 levels and vaginal ultrasound measurements of follicular diameter obtained every 2 or 3 days. Routine IVF or intracytoplasmic sperm injection (ICSI) was performed, as appropriate. ZIFT was performed 24 hours after ovum pick-up (OPU), and all patients received luteal support with progesterone.

Data on patient age and infertility-treatment-related variables were collected from the files. Ovarian stimulation characteristics, number of oocytes retrieved and number and quality of zygotes/embryos transferred were assessed and compared between the ZIFT cycle and the previous IVF/ICSI cycle and between those who conceived following the ZIFT cycle and those who did not. Embryos classification was based on the individual embryo scoring parameters according to pre-established definitions [14]. A top-quality embryo (TQE) was defined as seven or more blastomeres on day 3, equally-sized blastomeres and 20% fragmentation. Clinical pregnancy was defined as visualization of a gestational sac and fetal cardiac activity on transvaginal ultrasound.

Results are presented as means ± standard deviations. Differences in variables were statistically analyzed student’s t-test and chi-square test, as appropriate. Operating Characteristic (ROC) curve analysis was applied to all continuous quantitative variables. A p value of less than 0.05 was considered significant.

## Results

Forty seven patients undergoing their first ZIFT cycle were evaluated. Patients underwent their 10.2 ± 2.9 IVF cycle attempts during the study cycle (range, 6–19) and their mean age was 33.6 ± 4.9 years. The causes of infertility and the clinical characteristics of the IVF cycle which preceded the ZIFT cycle, in the all group and in those who did or did not conceive following the ZIFT cycle, are shown in Table 1.

**Table 1:** Comparison between the previous IVF cycles- related variables in patients who conceived and those who did not following ZIFT

	**All**	***Non pregnant***	***Pregnant***	**p values**
Number of cycles	47	35	12	
Patients’ age (years)	33.6 ± 4.9	34.2 ± 4.8	31.9 ± 5.0	ns
Cycle rank	10.2 ± 2.9	10.2 ± 3.0	9.5 ± 3.3	ns
Cause of infertility (% of all)				
Male	64.4%	63.6%	66.6%	ns
Oligo/anovulation	8.9%	9.1%	8.3%	ns
Others	26.6%	27.2%	25%	ns
COH protocol (% of all)				
Long agonist	63%	62.5%	63.6%	ns
Short agonist	11.5%	12.5%	9.1%	ns
Antagonist	25.5%	25%	27.2%	ns
Mean number of daily gonadotropin ampoules used	3.6 ± 1.5	3.8 ± 1.6	3.1 ± 1.1	p = 0.2
Peak E2 levels on day of hCG administration (pg/ml)	1984 ± 673	1951 ± 624	1937 ± 829	ns
Number of oocytes retrieved	11.1 ± 4.0	10.7 ± 4.4	11.9 ± 6.3	ns
Fertilization rate (%)	55 ± 21	50 ± 21	55 ± 33	ns
Number of 2PN	6.3 ± 4.3	5.6 ± 3.4	8.4 ± 6.1	p = 0.06
Total number of top quality	1.4 ± 1.9	1.6 ± 2.1	1.0 ± 1.5	NS
Embryos (TQE)				
Ratio of number of TQE to 2PN	24.5 ± 30.9	27.9 ± 33.7	13.9 ± 16.1	p = 0.2
Number of embryos transferred	2.9 ± 0.9	2.9 ± 1.0	3.0 ± 0.7	ns

When comparing the COH variable during the cycle preceding the ZIFT cycle, while there were no in-between group differences in the COH protocol used, the mean daily number of gonadotropin ampoules administered, peak estradiol levels, the number of oocytes retrieved, the number of TQE, or the number of embryos transferred, a trend toward a higher number of 2 pronuclei (PN) embryos was observed in those who conceived compared to those who did not (8.36 ± 6.07 vs 5.59 ± 3.36; p = 0.06), in the following ZIFT cycle. Moreover, a non-significant (p = 0.2) lower ratio of number of TQE to total 2PN embryos was observed in those who conceived (13.9 + 16.1 vs 27.9 + 33.7, respectively; p = 0.2).

Twelve clinical pregnancies (clinical pregnancy rate- 25.5%) were recorded following the ZIFT cycle, compared to none in the previous IVF cycle. However, it should be emphasized that the increased pregnancy rate in the ZIFT cycle is biased owing to the study design, which offered this approach to patients with RIF who had failed a previous IVF attempts.

When comparing the COH variable during the ZIFT cycle, there were no in-between group differences (Table 2), except for higher number of 2PN embryos in patients who conceived, compared to those who did not (8.45 ± 4.4 vs 5.9 ± 2.1; p < 0.01) and a non-significant higher fertilization rate (61 ± 17 vs 53 ± 18, respectively; p = 0.18).

**Table 2:** Comparison between the ZIFT cycles- related variables in patients who conceived and those who did not

	***Non pregnant***	***Pregnant***	**p values**
Number of cycles	35	12	
COH protocol (% of all)			
Long agonist	61.8%	60%	ns
Short agonist	23.5%	10%	ns
Antagonist	14.7%	30%	ns
Mean number of daily gonadotropin ampoules used	3.7 ± 1.5	3.0 ± 0.9	ns
Peak E2 levels on day of hCG administration (pg/ml)	2062 ± 962	1952 ± 628	ns
Number of oocytes retrieved	11.8 ± 4.5	13.3 ± 6.5	ns
Fertilization rate (%)	53 ± 18	61 ± 17	p = 0.18
Number of 2PN	5.9 ± 2.1	8.45 ± 4.4	p < 0.01
Number of 2PN transferred	4.5 ± 1.1	4.7 ± 0.9	ns

Receiver Operating Characteristic curve analysis of all the continuous quantitative variables did not reach statistically significant discriminatory value (Table 3), probably due to the small sample size, except for the number of 2PN achieved at the ZIFT cycle (>7, p < 0.05). Given the limitation above, it seems that patients who may benefit from ZIFT are young patients (≤31 yrs), who underwent ≤6 cycle attempts, yielding over eight 2PN embryos and ≤2 TQE, with low (≤0.4) ratio of number of TQE to total 2PN embryos. Moreover, in those destined for a ZIFT cycle, only those with >7 2PN embryo should undergo a transfer of at least five 2PN embryos.

**Table 3:** ROC analysis for COH variables in the previous IVF and the ZIFT cycles

**Variable**	**Cut off**	**Sensitivity**	**Specificity**	**p-value**
***Previous IVF cycle***				
Number of cycles	≤6	27.2	96.435	ns
Patients’ age (years)	≤31	58.3	71.4	ns
Mean number of daily gonadotropin ampoules used	≤3.5	90.9	41.9	ns
Peak E2 levels on day of hCG administration (pg/ml)	≤1656	54.5	64.5	ns
Number of 2PN	>8	54.5	90.6	ns
Total number of top quality	≤2	91.6	28.1	ns
Embryos (TQE)				
Ratio of number of TQE to 2PN	≤0.41	100.0	25.0	ns
Number of embryos transferred	>2	75.0	33.3	ns
***ZIFT cycle***				
Mean number of daily gonadotropin ampoules used	≤4	100	28.5	ns
Peak E2 levels on day of hCG administration (pg/ml)	≤2370	81.8	42.8	ns
Number of 2PN	>7	63.6	74.2	<0.05
Number of 2PN transferred	>4	75.0	48.5	ns

## Discussion

In the present study, patients with repeated IVF failures undergoing ZIFT, demonstrated an improved outcome with reasonable clinical pregnancy rate (PR) (25.5%). Moreover, although the hitherto published data does not provide any clear discriminatory criteria to aid selecting those RIF patients who will benefit from ZIFT, according to our experience, ZIFT should be offered to those patients with up to 6 IVF cycle failures, yielding over 8 2PN embryos with ≤2 TQE and that following the COH for the ZIFT cycle obtained at least 8 2PN.

Levran et al. [9] evaluated the efficacy of ZIFT on implantation and pregnancy rates in patients with repeated failure of implantation in IVF-ET cycles. Data on 70 patients who underwent 92 ZIFT cycles were compared to a control group consisting of patients with the same selection criteria who underwent an additional standard IVF-ET cycle during the same time period. The implantation and pregnancy rates (8.7%% and 34.2%, respectively) were significantly higher in the ZIFT group than in the control group. Moreover, in contrary to our observation, patients who persistently had low-quality embryos in previous IVF-ET cycles had relatively low PRs per transfer in the ZIFT cycles, as compared to those with better quality embryos.

Since ZIFT is supposed to improve the physiological environment for the growing zygote/embryo and to offer optimal conditions for embryonic development, those who might benefit from the procedure are patients with poor in-vitro embryo development. The observation by Levran et al. [9], together with their later study [10] that demonstrated significantly higher implantation and pregnancy rate in patients with RIF undergoing ZIFT as compared to extended culture with blastocyst stage transfer, actually contradict the aforementioned assumption.

In a recently published updated data from the same group, Weissman et al. [13] have summarized their experience with 280 laparoscopic ZIFT. They achieved 96 clinical pregnancies per attempt (34.3%) and 72 live births (25.7%). However, when referring to the issue of when and under which circumstances should ZIFT be offered to patients with RIF? they could not provide any prerequisite criteria. They concluded that whenever a thorough evaluation revealed no clear and treatable reason and the following therapeutic interventions i.e. mechanical irritation of the endometrium; preimplantation genetic screening; assisted hatching; co-culture; intracytoplasmic morphologically selected sperm injection; and blastocyst transfer failed to achieve pregnancy, ZIFT could be the next step.

According to our experience, ZIFT should be offered to young RIF patients (≤31 yrs), who underwent ≤6 cycle attempts, and who achieved over eight 2PN embryos with low (≤0.4) ratio of number of TQE to total 2PN embryos in their previous IVF-ET cycle. Moreover, in those destined for a ZIFT cycle, only those with >7 2PN embryo should undergo a transfer of at least five 2PN embryos, otherwise, a trans cervical ET should be undertaken.

Of notice, in contrary to Levran et al. [9,10], Aslan et al. [11] failed to demonstrate significant benefits from ZIFT procedure over IVF-ET in patients with repeated implantation failure, observation that was in agreement with Habana and Palter [12], who conducted a meta-analysis, demonstrating similar pregnancy and implantation rates in ZIFT and IVF-ET groups. These observations probably reflect a selection bias, resulting from the nowadays no established criteria for ZIFT.

## Conclusions

Repeated implantation failure patients who underwent ≤6 cycle attempts, yielding over eight 2PN embryos, with low (≤0.4) ratio of number of top-quality embryos to total 2PN embryos, may benefit from ZIFT. Further large prospective studies are needed to elucidate the role of this approach in patients with repeated IVF failures patients and to identify the specific characteristics of women that will aid both fertility specialists’ counseling and their patients in adjusting the appropriate infertility treatment.

## Abbreviations

COH: Controlled ovarian hyperstimulation; IVF-ET: In vitro fertilization-embryo transfer; OPU: Ovum pick-up; PN: Pronuclei; RIF: Repeated implantation failures; ZIFT: Zygote intrafallopian transfer; TQE: Top-quality embryo.

## Competing interests

The authors declare that they have no competing interests.

## Authors’ contributions

IG- Designed the study, assisted in writing the paper and edited it, proof read the paper and took part in discussions regarding the results. JL- Performed the operations, proof read the paper and took part in discussions regarding the results. GY- Performed a portion of the statistical evaluations, proof read the paper and took part in discussions regarding the results. JD- Performed the operations, proof read the paper and took part in discussions regarding the results. MB- Operated the laboratory part, proof read the paper and took part in discussions regarding the results. RO- The principal investigator, designed the study, performed the statistical evaluations, assisted in writing the paper and edited it in all its revisions. All authors read and approved the final manuscript.

## References

[B1] Penzias AS Improving results with assisted reproductive technologies: individualized patient-tailored strategies for ovulation induction Reprod Biomed Online 2004 9 43 46 10.1016/S1472-6483(10)62108-6 15257817

[B2] Killick S Ultrasound and the receptivity of the endometrium Reprod Biomed Online 2007 15 63 67 10.1016/S1472-6483(10)60693-1 17623538

[B3] Templeton A Morris JK Reducing the risk of multiple births by transfer of two embryos after in vitro fertilization N Engl J Med 1998 339 573 577 10.1056/NEJM199808273390901 9718375

[B4] Meldrum DR Silverberg KM Bustillo M Stokes L Success rate with repeated cycles of in vitro fertilization–embryo transfer Fertil Steril 1998 69 1005 1009 10.1016/S0015-0282(98)00083-1 9627284

[B5] Homburg S Meltcer J Rabinson S Scharf EY Anteby R Orvieto Is there a limit for the number of in-vitro fertilization cycles for an individual patient? Fertil Steril 2009 91 1329 1331 10.1016/j.fertnstert.2008.03.010 18468603

[B6] Margalioth EJ Ben-Chetrit A Gal M Eldar-Geva T Investigation and treatment of repeated implantation failure following IVF-ET Hum Reprod 2006 21 3036 3043 10.1093/humrep/del305 16905766

[B7] Urman B Yakin K Balaban B Recurrent implantation failure in assisted reproduction: how to counsel and manage. B. Treatment options that have not been proven to benefit the couple Reprod Biomed Online 2005 11 382 391 10.1016/S1472-6483(10)60847-4 16176683

[B8] Orvieto R Meltcer S Liberty G Rabinson J Anteby EY Nahum R A combined approach to patients with repeated IVF failures Fertil Steril 2010 94 2462 2464 10.1016/j.fertnstert.2010.03.057 20451192

[B9] Levran D Mashiach S Dor J Levron J Farhi J Zygote intrafallopian transfer may improve pregnancy rate in patients with repeated failure of implantation Fertil Steril 1998 69 26 30 10.1016/S0015-0282(97)00452-4 9457927

[B10] Levran D Farhi J Nahum H Royburt M Glezerman M Weissman A Prospective evaluation of blastocyst stage transfer vs. zygote intrafallopian tube transfer in patients with repeated implantation failure Fertil Steril 2002 77 971 977 10.1016/S0015-0282(02)03080-7 12009353

[B11] Aslan D Elizur SE Levron J Shulman A Lerner-Geva L Bider D Dor J Comparison of zygote intrafallopian tube transfer and transcervical uterine embryo transfer in patients with repeated implantation failure Eur J Obstet Gynecol Reprod Biol 2005 122 191 194 10.1016/j.ejogrb.2005.05.005 15950368

[B12] Habana A Palter SF Is tubal embryo transfer of any value? A meta analysis and comparison with the society for assisted reproductive technology database Fertil Steril 2001 76 286 293 10.1016/S0015-0282(01)01879-9 11476774

[B13] Weissman A Horowitz E Ravhon A Nahum H Golan A Levran D Zygote intrafallopian transfer among patients with repeated implantation failure Int J Gynaecol Obstet 2013 120 70 73 10.1016/j.ijgo.2012.07.018 23063734

[B14] Ziebe S Lundin K Janssens R Helmgaard L Arce JC for the MERIT Group Influence of ovarian stimulation with HP-hMG or recombinant FSH on embryo quality parameters in patients undergoing IVF Hum Reprod 2007 22 2404 2413 10.1093/humrep/dem221 17640944

